# Corrosion Inhibition Effect of Pyridine-2-Thiol for Brass in An Acidic Environment

**DOI:** 10.3390/molecules27196550

**Published:** 2022-10-03

**Authors:** Darshan Jayasinghe Karunarathne, Alireza Aminifazl, Tori E. Abel, Karen L. Quepons, Teresa D. Golden

**Affiliations:** Department of Chemistry, University of North Texas, 1155 Union Circle #305070, Denton, TX 76203, USA

**Keywords:** brass, copper alloy, corrosion, inhibitor, electrochemical impedance spectroscopy, X-ray photoelectron spectroscopy, Langmuir isotherm

## Abstract

In this study, the inhibitive performance of pyridine-2-thiol added to a corrosive solution was investigated for brass using potentiodynamic polarization, electrochemical impedance spectroscopy, and X-ray photoelectron spectroscopy. Electrochemical experiments were performed with different inhibitor concentrations in 0.5 M H_2_SO_4_ as the corrosive medium. For potentiodynamic polarization, i_corr_ values decreased significantly for the inhibited solutions in contrast with the uninhibited solution. Pyridine-2-thiol had an optimum inhibition concentration of 0.25 mM, giving an i_corr_ value of 1.8 µA/cm^2^ compared to 26 µA/cm^2^ for the blank solution. EIS data indicated that R_p_ and R_ct_ values increased substantially after the addition of the corrosion inhibitor and corrosion inhibition efficiencies of more than 85% was achieved for the majority of the inhibited solutions. Scanning electron microscopy showed defect free and less scale formation for the inhibited surface but the bare brass surface had larger amounts of scale formation. X-ray photoelectron spectroscopy and UV-vis spectroscopy was used to investigate surface chemical composition and inhibitor structural changes over time.

## 1. Introduction

Copper and its alloys are widely used in different industrial applications due to their excellent mechanical and chemical properties [1,2]. Brass, an alloy of copper and zinc, generally has a zinc content ranging from 30% to 39% with zinc percentages affecting the properties of the alloy. Brass is commonly used in marine applications, tubing, pipelines, and electronic equipment owing to its excellent thermal conductivity and corrosion resistance [3,4,5,6].

However, brass surfaces are susceptible to atmospheric corrosion over time, and this can lead to formation of large scale and corrosion products [3,4,7,8]. This can severely affect the thermal conducting ability of brass [9]. Therefore, cleaning the brass surface with appropriate pickling solution is important [3,10,11]. Generally, acids have been applied as pickling solutions with sulfuric acid one of the most common. During the pickling process, the brass surface is prone to damage by corrosion initiated in highly acidic environments [10,12,13,14,15]. When reacted with corrosive media, zinc tends to dissolve into the bulk solution and this process is known as dezincification [16,17]. Furthermore, copper also becomes oxidized because of the strong acidic nature of sulfuric acid, leading to severe damage. To reduce the damage from the pickling solutions, organic corrosion inhibitors are used extensively [3,4,18,19]. Organic corrosion inhibitors provide an efficient, practical, and cost-effective method to reduce damage to the metal surface from the pickling bath. Even though brass corrosion protection has been subjected to extensive research in different corrosive media, there are only a few reports which have focused on brass corrosion in sulfuric acid medium [20,21].

It is widely accepted that the inhibition effect of organic corrosion inhibitors strongly depends on the structure of the inhibitor organic molecule [21,22]. The inhibition process proceeds through a passive organic film formation on the metal surface. This phenomenon depends on several key factors such as surface charge of the metal surface, the structure of the corrosion inhibitor, and the corrosion medium. Compounds with heteroatoms (N, O, and S) and aromatic ring systems facilitates inhibitor film formation on the metal substrate, improving corrosion resistance [22,23,24]. Lone pair electrons on heteroatoms can form coordination bonds with transition metal atoms on the surface. Multiple researchers have used different types of organic corrosion inhibitors for brass corrosion inhibition in different media [25,26]. Recently, Zulfareen et.al. studied the corrosion inhibition effect of Mannich base compounds on brass in nitric acid medium and Radovanic et.al. investigated the brass corrosion inhibition in 3% NaCl in the presence of three environmentally friendly organic compounds [4,8]. Only a few studies have focused on the effect of organic inhibitors on brass corrosion in sulfuric acid media [13,19,20,21].

**Figure 1:**
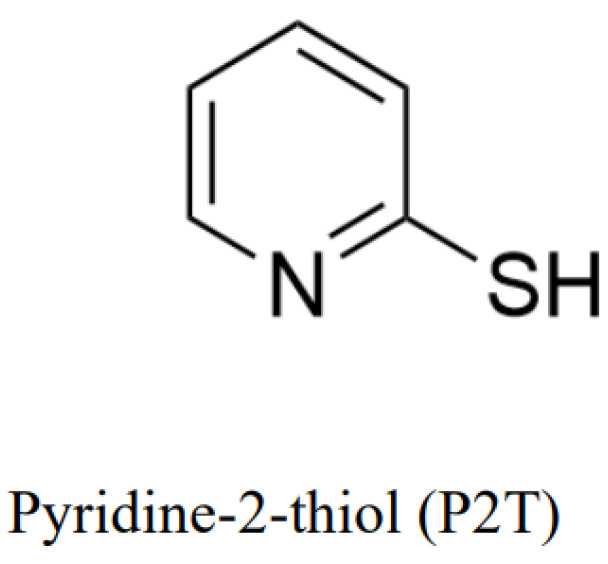
Molecular structure of pyridine-2-thiol (P2T), the inhibitor used for this study.

In this work, the corrosion inhibition effect is investigated for Pyridine-2-thiol (P2T) on brass in 0.5 M H_2_SO_4_ solution. This compound (Figure 1) consists of heteroatoms (N and S) and a ring structure that can interact with the substrate and enable the corrosion inhibition process.

Several studies have shown the corrosion inhibition effect of pyridine and thiol compounds on different metallic substrates [10,15,27]. It has been reported that a thiol compound can efficiently inhibit copper corrosion in acidic media by bonding with copper via S-Cu bond [10]. Furthermore, Kosari et.al. investigated the inhibitive ability of pyridine thiols on steel in different media and observed that P2T can significantly reduce the corrosion rate of the studied metals [28]. Recent studies by Tan et.al. suggests that disulfide compounds can be used as corrosion inhibitors for copper in acidic media and they observed that the studied disulfides gave excellent corrosion protection capability [29,30,31]. Nevertheless, only limited research has focused on studying these compounds as corrosion inhibitors for brass alloys and those have been mainly for chloride or nitric acid solutions. However, sulfuric acid is also a very common acid used for surface cleaning of brass in different industries. For the sulfuric acid medium, there could be a competitive adsorption process between the inhibitor molecules and sulphate ions on the brass surface, which is different from thiol adsorption from an organic solvent. Therefore, a study can provide insight into the adsorption mechanism of pyridine-2-thiol onto brass surface in sulfuric acid medium. The inhibitive performance of pyridine thiol on brass corrosion in 0.5 M H_2_SO_4_ medium is studied using several techniques. Electrochemical methods such as potentiodynamic polarization and electrochemical impedance spectroscopy (EIS) are used to study the corrosion inhibition mechanism of these compounds. Spectroscopic techniques like UV-vis spectroscopy and X-ray-photoelectron spectroscopy (XPS) are used to examine surface chemistry and composition.

## 2. Experimental

### 2.1. Materials and Solutions Preparation

Composition of the brass alloy used in this study is, Cu (67%) and Zn (33%). The corrosive media 0.5 M H_2_SO_4_ was made by diluting ACS grade sulfuric acid solution with DI H_2_O. Pyridine-2-thiol (97%) was purchased from Oakwood chemicals. Before each experiment the brass was polished using 320 to 1200 SiC grit paper. After polishing, the metal was ultrasonically washed with deionized water and ethanol then dried at room temperature.

### 2.2. Weight Loss Tests

Brass coupons (4.0 cm × 4.0 cm) were polished with SiC grit paper (320 to 1200) and then washed with distilled water and ethanol, followed by drying under a stream of nitrogen gas. Then, the initial weight of the dried coupons was recorded. Subsequently, samples were immersed in 1000 mL of 0.5 M H_2_SO_4_ at 25 °C for 72 h with different concentrations of P2T inhibitor. After 72 h immersion, coupons were removed from the solution and corrosion products were removed from the surface by hard brushing followed by sonication in water and ethanol. Finally, cleaned and dried sample weights were measured again to calculate the total weight loss. Each experiment was done in triplicate to ensure reproducibility and consistency. Average weight loss from the experiments was used to calculate the final weight loss and corrosion rates.

### 2.3. Electrochemical Testing

To evaluate the corrosion inhibition of P2T, potentiodynamic polarization and electrochemical impedance spectroscopy (EIS) were performed using a Potentiostat/Galvanostat instrument (PARSTAT 4000). All the electrochemical tests were performed using a conventional three electrode system with the brass alloy (1 cm^2^) as the working electrode and saturated calomel electrode (SCE) with a lugging capillary as the reference electrode. A platinum mesh was used as the counter electrode. Before performing potentiodynamic polarization and electrochemical impedance spectroscopy, open circuit potential (OCP) was measured for 3600 s to obtain a stable OCP value. Potentiodynamic polarization was performed from ±250 mV vs. OCP with a scan rate of 0.167 mV/s. The corrosion potential, E_corr_, was determined as the point of intersection of the anodic and cathodic polarization branches. To obtain an estimation of the corrosion current, i_corr_, for the inhibitor samples, a horizontal line was drawn at the E_corr_ value and another horizontal line was drawn 100 mV cathodic from E_corr_. A slope line was drawn from the 100 mV meeting point on the cathodic branch to intersect with the E_corr_ line. The point of intersection was taken as the value of i_corr_. EIS measurement was carried out in the frequency range of 0.01 Hz and 10,000 Hz with an amplitude of ±10 mV. EIS data from different samples were further analyzed by equivalent circuit modeling and data fitting using ZView software.

### 2.4. Atomic Absorption Spectroscopy (AAS)

In order to examine the long-term effect of P2T on brass, a 2 cm × 2 cm square sample was immersed in 0.5 M H_2_SO_4_ solution at 25 °C only or with 0.25 mM addition of the inhibitor. Standard calibration curves for copper and zinc were first completed within the measurement range of interest. Copper and zinc concentrations of the immersion solution was measured using PerkinElmer AAnalyst 300 Atomic Flame Absorption Spectrometer in intervals of 2, 4, 6, 8, 12, 16, 20, 24, and 48 h.

### 2.5. Scanning Electron Microscopy (SEM)

Brass specimens were polished with SiC grit papers, cleaned and cut into 1 cm × 1 cm coupons. Samples were then immersed in solutions of 0.5 M H_2_SO_4_ with 0.25 mM of inhibitor concentration. Another sample was immersed in the acid solution without adding any inhibitor. After 24 h of immersion, samples were taken out, dried and the surface of the brass specimen was analyzed using a FEI Quanta 200 SEM instrument.

### 2.6. X-Ray Photoelectron Spectroscopy (XPS)

0.5 cm × 0.5 cm brass samples were immersed in 0.5 M H_2_SO_4_ solution with 0.25 mM of P2T. After 6 h of immersion, the sample was taken out, washed with distilled water to remove loosely bound species and dried using a nitrogen gas stream before further analysis. X-ray photoelectron spectra (XPS) were obtained using a PHI 5000 Versa probe X-ray photoelectron spectroscopy instrument equipped with monochromatic 1486.6 eV Al Kα radiation source. Survey spectra of samples were taken using a pass energy of 183.5 eV and core level spectra were performed using a pass energy of 23.5 eV. XPS measurements were carried out under the pressure of 10^−7^ torr or lower. Casa XPS software was used to analyze obtained spectra and spectral calibration was done using C 1s peak at 284.8 eV.

### 2.7. UV-Vis Spectroscopy

UV-vis spectroscopy was run for the immersion solutions. Aliquots of the immersion solutions with inhibitor were taken at 0, 12, 16, 20, 24, and 48 h and put into a 1 cm^2^ cuvette. Scans were run with an HP-8453 UV-vis instrument from 200 to 450 nm wavelength range at the different immersion times to see any structural changes for pyridine thiol.

## 3. Results and Discussion

### 3.1. Weight Loss Tests

The corrosion rates (*CR*) and inhibition efficiencies (*IE*) were calculated from weight loss data using the following equations (Equations (1) and (2)). Table 1 lists the calculated values for the blank solution and different concentrations of P2T.

(1) $$CR=\frac{W_{i}-W_{f}}{AT}$$

(2) $$IE\%=\frac{CR_{blank}-CR_{inh}}{CR_{blank}}\times 100$$

where *W_i_* (g) is the initial weight of the coupons, *W_f_* (g) is the weight after the immersion tests, *A* (m^2^) is the surface area and *T* (h) is the immersion time. In Equation (2), *CR_inh_* and *CR_blank_* are the corrosion rates with and without the inhibitor in solution.

As shown in Table 1, addition of P2T into the immersion solution decreases the corrosion rates. Nevertheless, at lower inhibitor concentrations, inhibition efficiency is relatively low, 0.05 and 0.10 mM concentrations having average inhibition efficiencies of 4.5 and 11.7%, respectively. Higher concentrations of the inhibitors show acceptable level of inhibition efficiencies, where 0.50 mM demonstrates 44.5%. Gravimetric data proves that adding P2T into 0.5 M H_2_SO_4_ reduces the corrosion process, and other studies show that adding corrosion inhibitors to corrosive media reduces the weight loss of the metal by forming a protective film [32,33].

### 3.2. Atomic Absorption Spectroscopy (AAS)

**Figure 2:**
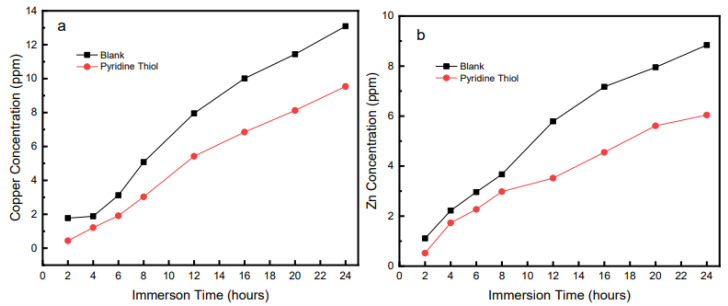
Copper (**a**) and Zinc (**b**) concentration (ppm) present in 0.5 M H_2_SO_4_ solution with respect to immersion time for 0.5 mM P2T obtained by atomic adsorption spectroscopy measurements.

Atomic absorption spectroscopy is an effective technique to investigate the corrosion protection of various corrosion inhibitors [34,35]. Since the corrosion process of brass consists of oxidation of both zinc and copper, analyzing the corrosive solution for Zn and Cu ions after exposure could give a better understanding of the overall corrosion process and effect of P2T over time. Figure 2 shows the zinc and copper concentrations of aliquots from the immersion solution with respect to time.

In the presence of pyridine thiol, the copper concentration in the immersion solution is lower than that of the uninhibited solution for all immersion times in 0.5 M H_2_SO_4_. The zinc concentration also follows a similar trend but with increasing immersion time both copper and zinc concentration increase in both the inhibited and uninhibited solutions. It is important to notice that even with higher immersion times up to 24 h, the P2T solution has lower copper and zinc concentrations with respect to the uninhibited solutions. This shows that P2T can provide excellent corrosion protection in acidic solutions for certain time periods. In the presence of a strong acid like sulfuric acid both copper and zinc become oxidized and diffuse into the solution, but when pyridine thiol is present, a reduction in oxidation and subsequent diffusion of ions into the solution is inhibited by the thin P2T film on the surface of the metal.

**Figure 3:**
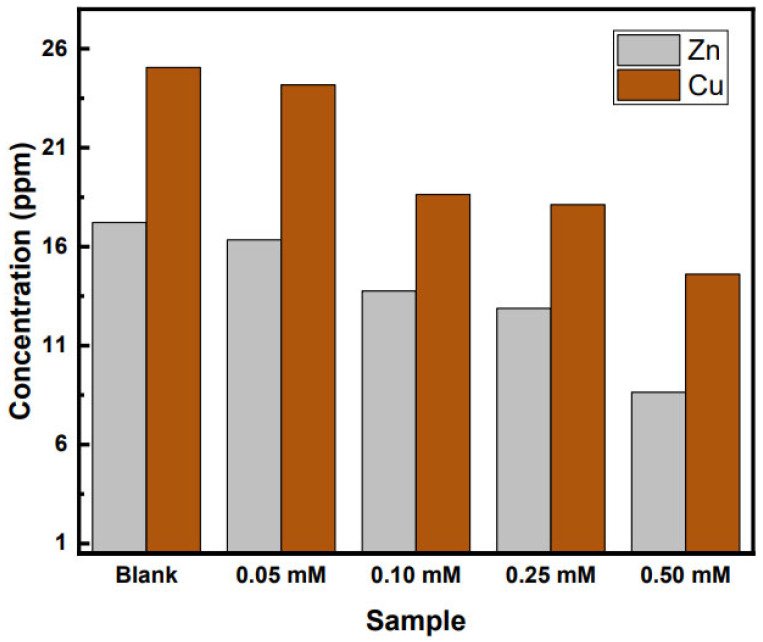
Copper and Zinc concentrations after an immersion time of 48 h in 0.5 M H_2_SO_4_.

In order to see the effect of inhibitor concentrations, atomic absorption analysis was performed after 48 h for different inhibitor concentrations. Figure 3 shows the copper and zinc ion concentrations in 0.5 M H_2_SO_4_ after 48 h of immersion.

Final copper ion concentration for the uninhibited solution was 24.5 ppm, but lower in all the inhibitor solutions. Similarly, the zinc ion concentration in the uninhibited solution was 16.7 ppm, but decreased with increasing inhibitor concentration. Inhibition efficiencies were calculated for both ions in different concentrations. (see Supplementary Materials Table S1 and S2) Largest inhibition efficiency is seen for a 0.50 mM concentration of inhibitor (51.3% for Zn and 42.5% for Cu). These values have the same trend as the gravimetric data. At lower inhibitor concentrations, there might not be enough to cover the whole metal surface with a protective film. Furthermore, inhibitor desorption from the surface can also affect this behavior [36]. After 48 h of immersion, higher concentrations of inhibitor show lower copper and zinc ion concentrations in solution compared to the blank uninhibited solution.

### 3.3. Scanning Electron Microscopy (SEM)

**Figure 4:**
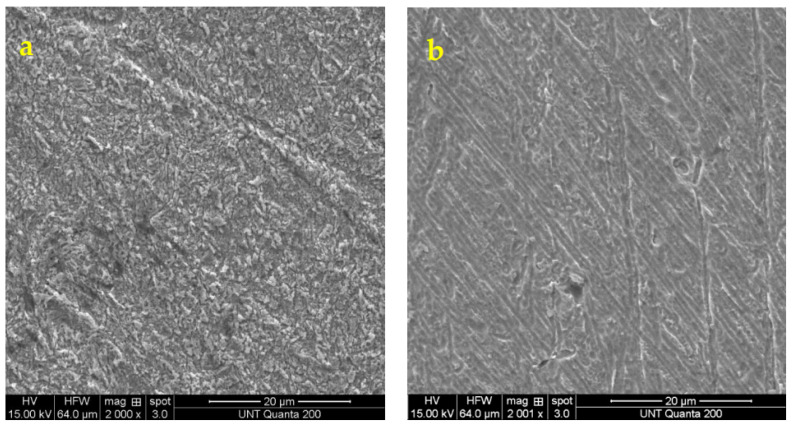
SEM images of brass after immersion in 0.5 M H_2_SO_4_ solution (**a**) without any inhibitor for 24 h, 2000× magnification and (**b**) with 0.25 mM of inhibitor for 24 h, 2000× magnification.

The SEM images of a brass specimen after immersion for 24 h in 0.5 M H_2_SO_4_ solution with and without the inhibitor are shown in Figure 4. In Figure 4a, the surface has roughened after immersion in 0.5 M H_2_SO_4_ for 24 h indicating corrosion products present on the brass surface due to the aggressive corrosion attack by sulfuric acid on the unprotected surface. These corrosion products were further studied with XPS. In contrast, the inhibited brass samples only have a minor amount of product formation. The larger agglomerated corrosion products which are visible in the uninhibited solution is absent in the P2T inhibited brass samples. Surface looks smoother and uniform in comparison with the blank solution. This further confirms the adsorption of P2T and formation of the protective film on the metal surface providing protection from aggressive acidic corrosion attack by sulfuric acid.

### 3.4. Potentiodynamic Polarization

**Figure 5:**
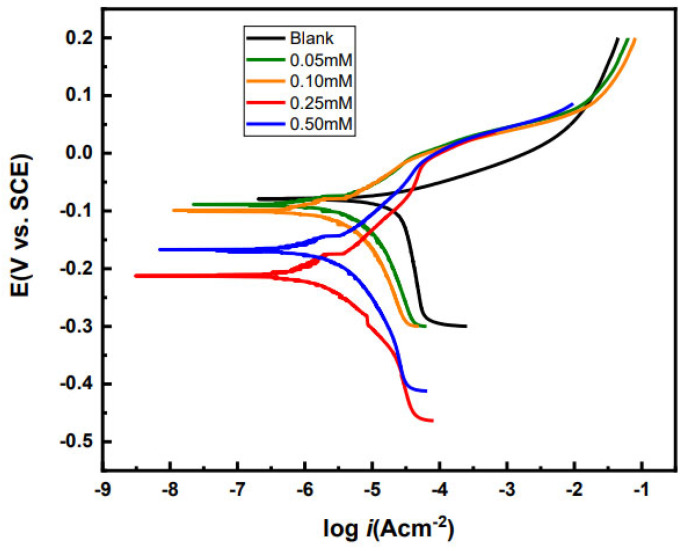
Potentiodynamic polarization curves of brass samples with and without corrosion inhibitors in 0.5 M H_2_SO_4_ at 25 °C.

Electrochemical experiments were done to explore the more immediate effects of the inhibitor interaction with the brass substrate. Potentiodynamic polarization curves of brass with different concentrations of P2T in 0.5 M H_2_SO_4_ at 25 °C are shown in Figure 5. Important electrochemical parameters including corrosion potential (*E_corr_*), corrosion current density (*i_corr_*), anodic and cathodic slopes (βa and βc) have been calculated and listed in Table 2. Additionally, surface coverage (Ɵ) and inhibition efficiency (µ) have been calculated using the following equations (Equations (3) and (4)) [37].

(3) $$Ɵ=\frac{icorr_{blank}-icorr_{inh}}{icorr_{blank}}$$

(4) µ%=Ɵ×100

For the surface coverage equation *icorr_blank_* represents uninhibited corrosion current density and *icorr_inh_* is inhibited corrosion current density. In 0.5 M H_2_SO_4_ medium the cathodic reaction of brass corrosion is oxygen reduction (Equation (5)) [12].

½ O_2_ (g) + 2H^+^ (aq) + 2e^−^ → H_2_O (5)

Anodic dissolution is a complex process for brass in 0.5 M H_2_SO_4_ medium and this process includes dissolution of both Cu and Zn (Equations (6)–(8)). Even though zinc has a higher tendency to become oxidized, copper can oxidize via a twostep process (Equations (6) and (7)) [15,38,39].

Cu (s) → Cu^+^ (ads) + e^−^   (fast step) (6)

Cu**^+^** (ads) → Cu^2+^ (aq) + e^−^  (slow step) (7)

Zn (s) → Zn^2+^ (aq) + 2e^−^ (8)

As shown in Figure 5. the brass sample without an inhibitor shows a tafel type behavior in the anodic branch of the scan, while the cathodic polarization exhibits a limiting current due to dissolved oxygen reduction at the electrode surface. For the anodic curve of the blank sample, there is a rapid increase in current resulting in a lower anodic tafel slope value around 40 mV/dec compared to the inhibitor scans, which has also been seen by other researchers for copper and copper alloy corrosion in sulfuric acid solutions. There are changes seen for the scans with the inhibitors. After the addition of P2T, cathodic limiting current is slowed and cathodic tafel slope values for the inhibited solutions are lower compared to the uninhibited solution. This suggest that P2T addition slowed down the oxygen reduction reaction probably by covering the metal surface, limiting access to cathodic active sites. Additionally, for the inhibited solutions the anodic curve is missing the rapid increase in current that is seen for the uninhibited solution. There is a small passivation region present ~100 to 200 mV contained in the anodic curve for the inhibitor solutions indicating resistance to anodic dissolution.

Table 2 list the calculated electrochemical parameters (*i_corr_*, *E_corr_*, β_c_, and β_a_) from the potentiodynamic polarization curves. The corrosion potential (*E_corr_*) shifts to more negative values with addition of the inhibitor, indicating cathodic type inhibition behavior. The largest shifts for the corrosion potential (132 and 87 mV compared to the blank) are seen for the experimented inhibitor concentrations 0.25 and 0.50 mM. If the *E_corr_* shift is larger than 85 mV, the inhibitor is considered a cathodic type inhibitor and if the shift is smaller than 85 mV, the inhibitor is considered a mixed type inhibitor [40,41,42]. Since at higher P2T concentrations, the shift is larger than 85 mV, P2T behaves as a cathodic inhibitor. Additionally, the *E_corr_* value negative shifts indicate an increase in cathodic inhibition. With increasing inhibitor concentration, *i_corr_* values decrease and inhibition efficiency increases. The 0.25 mM P2T inhibitor solution has the lowest *i_corr_* and inhibition efficiencies, while these values increase at a higher inhibition concentration of 0.5 mM. This indicates an optimum concentration to achieve best inhibition. Other research groups have also seen observed this behavior for various corrosion inhibitors [15,43].

From the potentiodynamic polarization data, it is evident that inhibition efficiencies calculated from gravimetric analysis and potentiodynamic polarization are different. Potentiodynamic polarization data shows higher inhibition efficiencies for pyridine-2-thiol. This phenomenon has been observed by other researchers as well [40]. The two methods give different corrosion protection values since they measure two different mechanisms. Electrochemical measurement gives the corrosion rate at a specific time under accelerated corrosion conditions and specifically provides an idea about what is happening at the electrode-solution interface at that time. Contrastingly, weight loss measurements present the average corrosion rate over a long period of time and assume uniform corrosion across the metal surface.

### 3.5. Electrochemical Impedance Spectroscopy (EIS)

**Figure 6:**
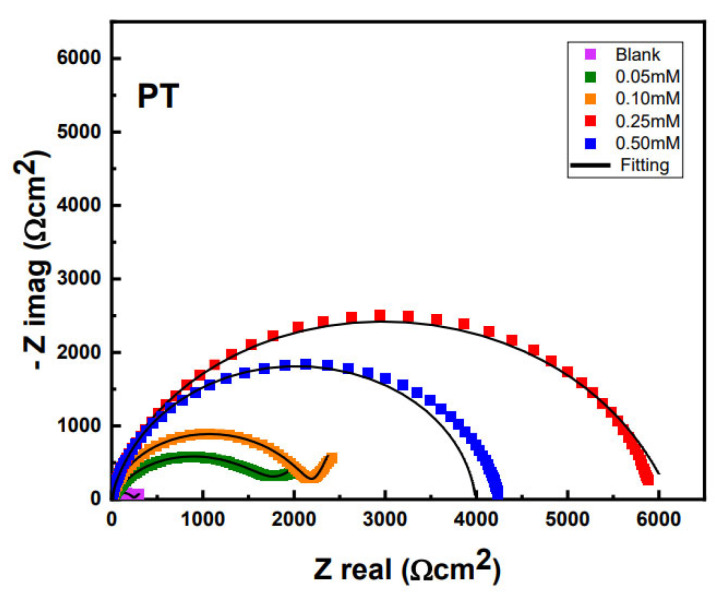
Nyquist plots of brass samples with and without different concentrations of corrosion inhibitors in 0.5 M H_2_SO_4_.

To have a better understanding of the inhibition effect on brass corrosion in 0.5 M H_2_SO_4_ medium, electrochemical impedance spectroscopy was performed with and without the P2T inhibitor. Figure 6 presents the corresponding Nyquist plots of the inhibitor at varying concentrations in the solution. As shown in Figure 6, the Nyquist plot demonstrates a capacitive loop at higher frequencies and a straight line at lower frequencies in blank solution and in the presence of lower concentrations of P2T. High frequency semicircle is generally associated with the charge transfer resistance and the straight line at lower frequency is due to the Warburg impedance cause by diffusion of chemical species either from or into the bulk solution [31,38]. In this case, dissolved oxygen diffuses to the brass surface or metal ions diffuse from the brass surface to the solution causing Warburg impedance [29,30,31]. With increasing concentration of P2T, the Warburg line disappears, indicating a uniform inhibitor layer formation on the electrode surface hindering the diffusion processes. With increasing inhibitor concentration, the radius of the semicircle increases. This can be attributed to the larger charge transfer resistance caused by the adsorbed inhibitor layer on top of the brass surface [10,15,19]. Even though increasing inhibitor concentrations result in larger semi circles, the 0.25 mM concentration has the largest semicircle which indicates the best corrosion resistance, while higher concentrations than 0.25 mM have a decrease in corrosion resistance. Ebrahimzadeh and N.Wei has also seen similar behavior with pyridine based compounds using different metals [15,43]. In lower concentrations of the inhibitor, the inhibitor layer coverage may be too low to protect the entire surface from the corrosive medium. However, for higher concentrations than 0.25 mM, the inhibitor molecules may agglomerate, leading to loosely bound molecules on the surface which may not give enough corrosion resistance compared to the optimum inhibitor concentration (0.25 mM) where a tightly bound, uniform coating can form.

**Figure 7:**
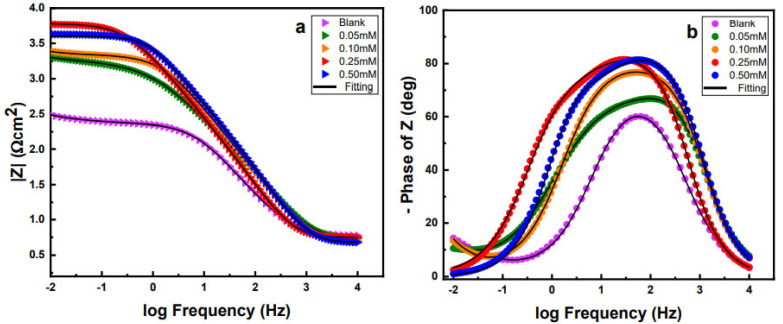
Bode magnitude (**a**) and Phase angle (**b**) plots of brass samples with and without different concentrations of corrosion inhibitors in 0.5 M H_2_SO_4_.

Figure 7 shows the Bode magnitude (a) and phase angle (b) plots for the brass in 0.5 M H_2_SO_4_ with and without the inhibitor. For the Bode magnitude plot, the total impedance (|z|) at lower frequency is significantly higher in the presence of P2T in comparison with the uninhibited solution. This indicates that the inhibitor provides corrosion protection to the brass surface for a wide range of inhibitor concentrations. Furthermore, 0.25 mM of P2T gives the largest impedance confirming the better inhibition effect at that concentration. Phase angle plots have a higher and wider phase values in contrast with the blank solution which suggests better corrosion protection. Furthermore, in the presence of inhibitor the phase angle plot exhibits a two-time constant behavior and at optimum concentrations phase angle values approach close to 80° and shows wider phase angle in wide range of frequency demonstrating better corrosion inhibition.

**Figure 8:**
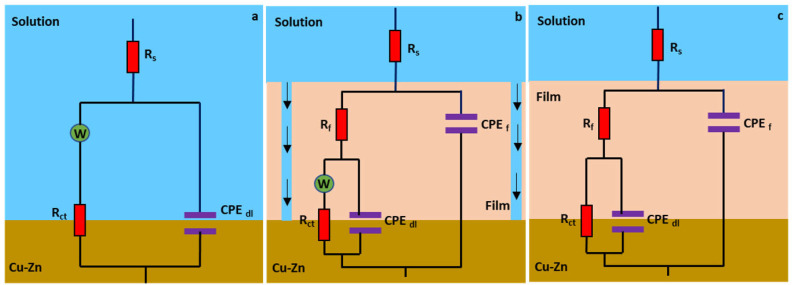
Equivalent circuit models used to fit electrochemical impedance data for (**a**) the blank solution, (**b**) 0.05 and 0.10 mM concentrations of inhibitor solutions showing Warburg impedance, and (**c**) 0.25 and 0.50 mM concentrations of inhibitor solutions.

The impedance data was modeled with Zview software, to have a better understanding of the inhibition mechanism. Three different equivalent circuits were used and shown in Figure 8 for (a) the blank solution, (b) the inhibited solutions with Warburg impedance and (c) the inhibited solutions without Warburg impedance. R_s_, R_f_, R_ct_ and W represents solution resistance, resistance of the film, charge transfer resistance and Warburg impedance, respectively, and the fitted chi-squared values were less than 0.001 for each circuit. Instead of using a true capacitor, a constant phase element was used to fit the data and CPE_f_ is the capacitance of the film and CPE_dl_ is the double layer capacitance. These components reflect film capacitance (C_f_) and double layer capacitance (C_dl_). The impedance of *CPE* is represented by the following equation (Equation (9)),

(9) $$ZCPE=\frac{1}{Yo\;\left(jw\right)n}$$

where *Yo* is the magnitude of the *CPE*, *j* is the imaginary root and *w* is the angular frequency. The value *n* represents the deviation parameter from an ideal capacitor.

The values for C_dl_ and C_f_ can also be calculated using a formula proposed by Brug et al. [44] and are listed in the Supplementary Materials as Table S3. The C_dl_ and C_f_ values follow the same trend as the EIS data in Table 3 which are the calculated values using the equivalent circuit modeling.

From equivalent circuit fitting data, it is evident that addition of the corrosion inhibitor increases both R_f_ and R_ct_ values. In the blank solution, R_ct_ value for the brass without inhibitor is about 224 Ω cm^2^ but when the concentration of P2T is at 0.25 mM, R_ct_ reaches 4238 Ω cm^2^ showing significant improvement in corrosion inhibition. The film resistance (R_f_) also increases with the addition of P2T in the corrosive solution and a maximum value of 1691 Ω cm^2^ can be seen at 0.25 mM concentration, before decreasing at higher concentration of 0.5 mM. The 0.25 mM concentration fitting values indicate the best corrosion resistance, while higher concentrations than 0.25 mM have a decrease in corrosion resistance. This follows the same trend as potentiodynamic polarization data and further clarifies that there is an optimum adsorption concentration for the P2T inhibitor. The same type of trend is seen for the calculated C_f_ and C_dl_ values.

### 3.6. Adsorption Isotherms

Adsorption isotherm studies were conducted in order to study the interaction mechanism between the surface and the inhibitor molecules. There are two main types of interactions involved with inhibitor adsorption onto a metallic surface, namely physisorption and chemisorption. Physisorption refers to the physical adsorption between two charged species, in this case the charged metal surface and the protonated pyridine thiol molecule. Chemisorption is where coordination bonding occurs between inhibitor molecules and the metal surface. Generally, physisorption process involves both electrically charged metal surface and charged species in the bulk solution. In this solution negatively charged SO_4_^2−^ anions are abundant and these anions compete with the pyridine thiol for adsorption on the metal surface. When sulfate ions adsorb into the brass surface, these ions can make the surface negatively charged. Nitrogen center in the pyridine thiol molecule can become protonated in the sulfuric acid medium and adsorb to the negatively charged brass surface through electrostatic attraction between the protonated nitrogen and negatively charged sulfates. Chemisorption involves any type of bond formation between the surface and the inhibitors, and it can be in the form of dative bonds or covalent bonds. Anodic dissolution of brass involves three major reactions as shown in Equations (6)–(8). Thiol can make a covalent bond with the copper surface (Cu-S) and this a well-known phenomenon that is widely seen in other similar studies as well. The main contribution to the chemisorption process should be this covalent bond formation. Another possibility is that zinc ions form a complex with the pyridine thiol molecule and provide surface coverage.

**Figure 9:**
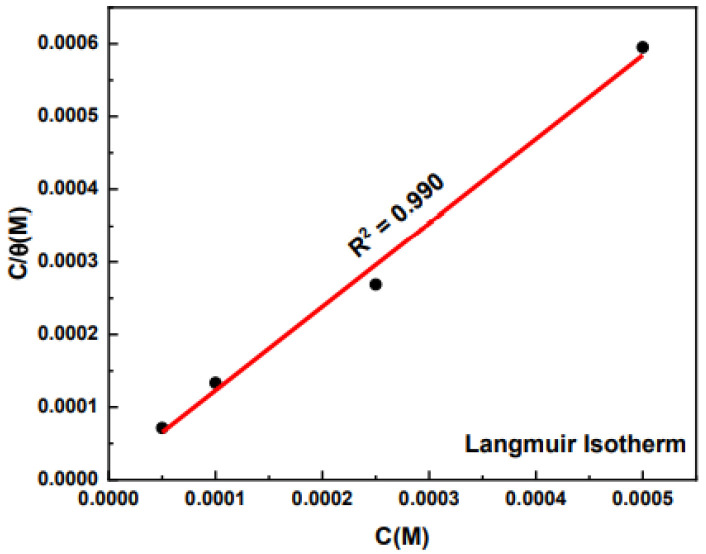
Langmuir adsorption isotherm fitting using the potentiodynamic polarization data at 25 °C.

Several isotherm models were used to fit the surface coverage data which was obtained from the potentiodynamic polarization experiments run at 25 °C (298 K). Langmuir, Temkin, Frumkin, Florry-Higgins, El-Awady, and Fruenlich isotherms were all used to fit the experimental data to find best fit. Generally larger fitting correlation coefficient values (R^2^) give the best fitting adsorption model. For the P2T adsorption data, the Langmuir isotherm (R^2^ = 0.99) (Figure 9) gave the best fitting values. Adsorption data fitting and equations for the other models can be found in the Supplementary Materials (Figure S1).

The Langmuir isotherm model has been used in several studies to determine the adsorption mechanism of organic corrosion inhibitors onto metallic surfaces as well as the thermodynamic parameters of the adsorption process [45,46,47]. According to this isotherm, the surface coverage (Ɵ) relates to the inhibitor concentration as shown in the following equation:

(10) $$\mathrm{Langmuir}\;\mathrm{isotherm}:\;\;\frac{C}{Ɵ}=\frac{1}{K_{ads}}+c$$

where *K_ads_* is the equilibrium constant for the adsorption process, C is the concentration of the corrosion inhibitor. As seen in the adsorption isotherm, a strong correlation (R^2^ = 0.99) is obtained for the Langmuir isotherm and that further confirms the adsorption mechanism of P2T to brass surface. The adsorbed molecules do not have any interactions with other adsorbed species and most likely there is a formation of only a monolayer on the metal surface. Thiol compounds are well known to form self-assembled monolayers with metallic surfaces like gold and copper, forming Cu-S bonds. The calculated equilibrium constants for adsorption (*K_ads_*) of pyridine thiol onto brass surface is 1.28 × 10^5^ L/mol. From the equilibrium constant, Gibbs free energy of adsorption process (∆G^o^_ads_) was calculated using following equation [13,14,15,23,24,25,26,27].

∆G^o^_ads_ = − RT ln (55.5*K_ads_*) (11)

where R is the ideal gas constant, T is the absolute temperature and 55.5 is the concentration of water in solution [15]. Using this method Gibbs free energy values of −39.08 kJ/mol was obtained for P2T. In this case, Gibbs free energy is a negative value which implies that the inhibitor adsorption to the brass surface is a spontaneous process. It is widely accepted that if the ΔG value is less negative than −20 kJ/mol, the adsorption proceeds through physisorption and if ΔG is more negative than −40 kJ/mol it is predominantly a chemisorption process. Since the value for P2T is −39.08 kJ/mol, this indicates the process leaning more towards chemisorption. This implies that the chemisorption process is proceeding through bonding between pyridine thiol and the brass surface. However, some physisorption is still possible. XPS can be used to further elucidate the surface bonding occurring during adsorption.

### 3.7. X-ray Photoelectron Spectroscopy (XPS)

**Figure 10:**
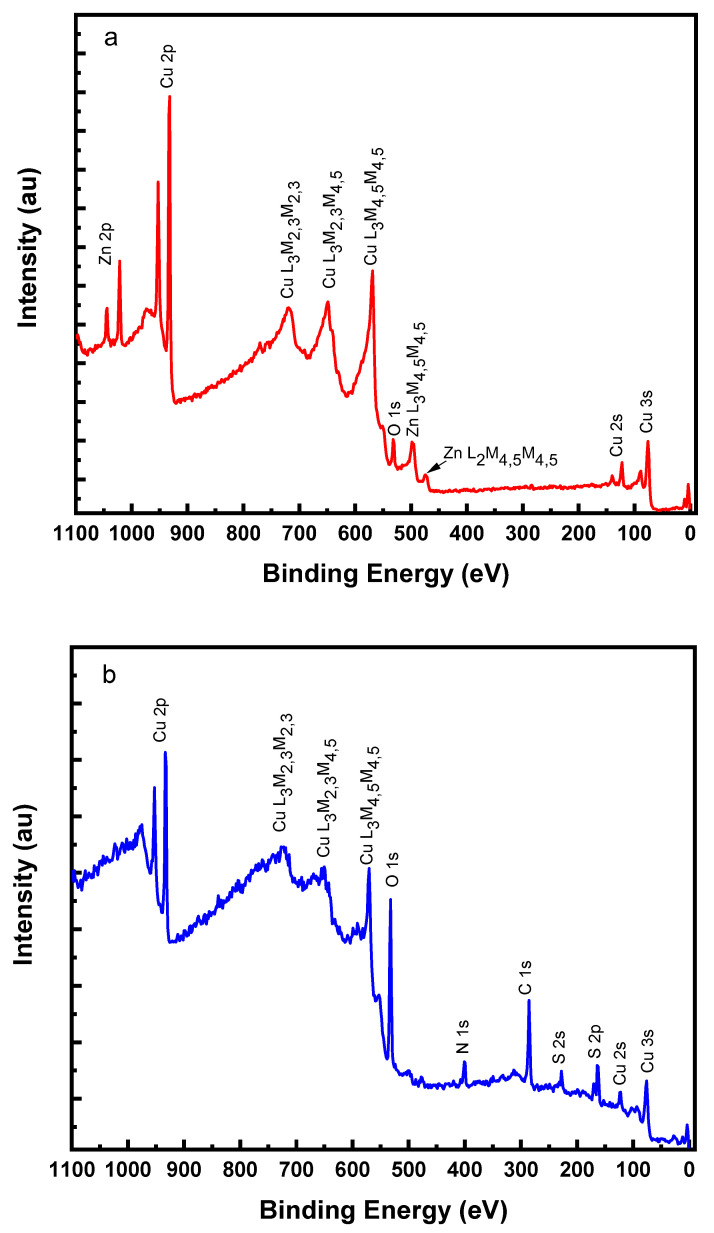
Survey spectrum of (**a**) sputter cleaned brass sample and of (**b**) 0.25 mM P2T inhibitor treated brass sample in 0.5 M H_2_SO_4_ for 6 h.

X-ray photoelectron spectroscopy was used in order to obtain details about the surface composition of pyridine thiol on the brass substrate. Figure 10a represents the survey spectrum of the pure brass sample that was cleaned using Ar^+^ sputtering to remove adventitious carbon contamination.

The spectrum consists of typical Cu 2p, Cu 2s, Cu 3s and Zn 2p photoelectron lines and also intense XPS excited Auger lines of Cu L_3_M_4,5_M_4,5_, Cu L_2_M_2,3_M_4,5_, Cu L_3_M_2,3_M_2,3_, Zn L_2_M_4,5_M_4,5_, and Zn L_3_M_4_,_5_M_4_,_5_ lines. The survey spectrum of pyridine thiol treated brass sample (Figure 10b) shows Cu signals including Cu 2p, 2s, 3s as well as the XPS excited Auger (Cu L_3_M_4,5_M_4,5_, Cu L_2_M_2,3_M_4,5_ and Cu L_3_M_2,3_M_2,3_) signals but no signals originating from Zn. Furthermore, C 1s, S 2s, S 2p, O 1s and N 1s signals can also be seen in the survey scan of P2T treated brass sample [48,49].

In order to obtain more information about the surface layer of the inhibited brass sample, elemental scans were performed, and peaks were deconvoluted using CASA XPS software.

**Figure 11:**
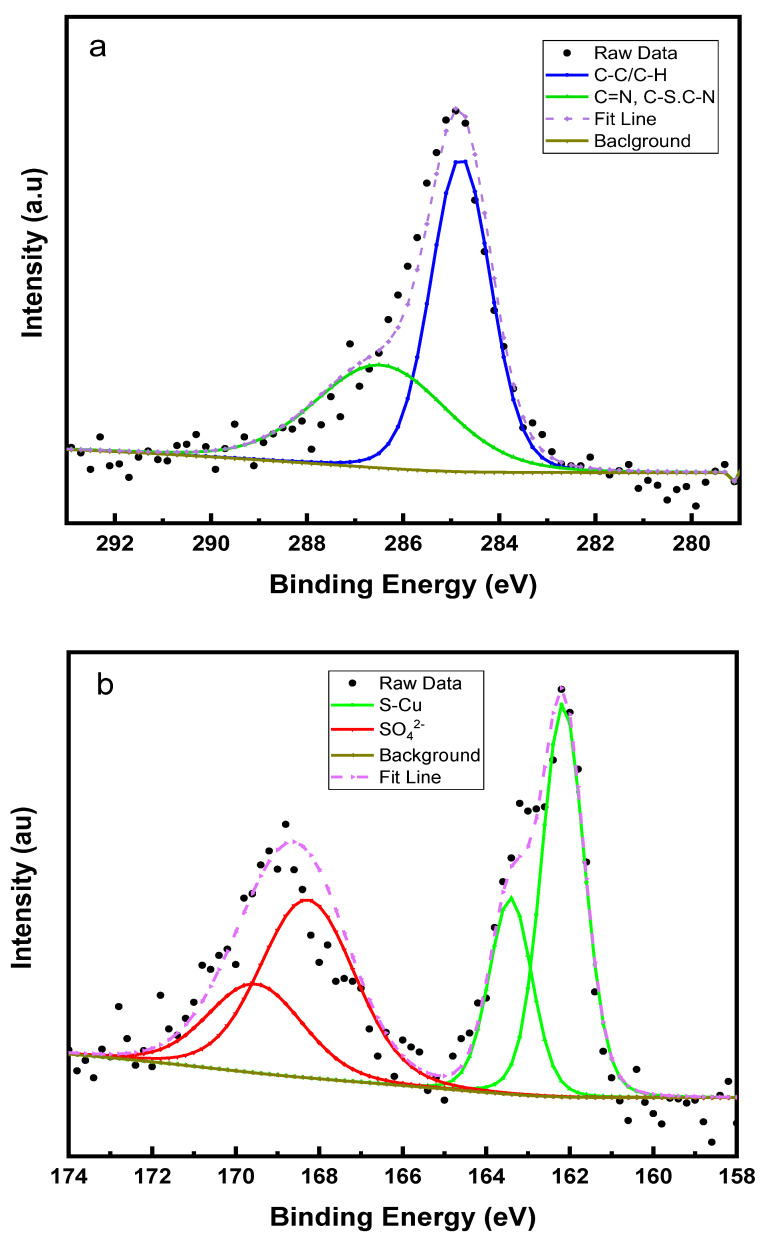
(**a**) C 1s and (**b**) S 2p spectra of P2T treated brass sample.

Figure 11a shows the C 1s spectrum, and it has two peaks at 284.8 and 286.5 eV corresponding to different carbon atoms in the heterocyclic ring. The peak at 284.8 represents the C=C and C-H bonding in the ring and 286.5 eV is related to carbon atoms connected to nitrogen and sulfur atoms. Figure 11b shows the S 2p spectrum with two spin-orbit split peaks. Typically, the S 2p peak has two spin-orbit components (S 2p_3/2_ and S 2p_1/2_) with a 1.2 eV difference and 1:2 peak area ratio and these two peaks represents two different sulfur bonding patterns on the brass surface [50]. The peak with binding energy of 162.2 eV (S 2p_3/2_) corresponds to the S-Cu bonding formation and the other peak at higher binding of 168.3 eV (S 2p_3/2_) can be assigned to adsorption of sulfate from the bulk solution.

**Figure 12:**
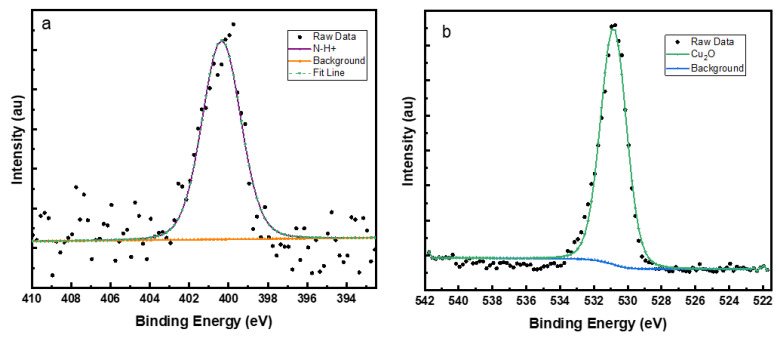
(**a**) N 1s and (**b**) O 1s XPS spectra of P2T treated brass sample.

**Figure 13:**
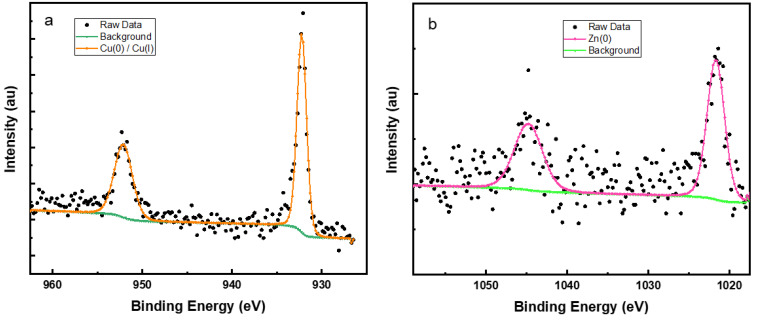
(**a**) Cu 2p and (**b**) Zn 2p XPS spectra of P2T treated brass sample.

This shows that the inhibitor molecules attach through a S-Cu bond to give a stable inhibitor layer coverage on the brass surface. The N and O XPS spectra are shown in Figure 12a,b. The N 1s spectrum consists of a single peak at binding energy of 400.3 eV and can be assigned to the protonated nitrogen atom in the pyridine ring [51,52]. There is no peak visible for N-Cu bond formation around 399 eV which confirms that all the nitrogen atoms are in the protonated form, since the pH of the corrosive medium is less than 1 [53,54]. The O 1s peak (Figure 12b) has only one component at a binding energy around 530.8 eV which can be attributed to the formation of Cu_2_O [55]. There is no peak around 529.7 eV for the formation of CuO. This indicates that adsorption of pyridine thiol blocks oxidation of Cu^+^ to Cu^2+^ efficiently. Additionally, this is confirmed for the Cu 2p peaks (Figure 13a) which show two spin-orbit doublet components (Cu 2p_3/2_ and Cu 2p_1/2_) at 932.2 and 952.1 eV, respectively. Cu 2p_3/2_ peak at 932.2 eV can be assigned to Cu_2_O and there are no satellite peaks that are characteristic of CuO formation [56].

The Zn XPS signal (Figure 13b) is not very clear and has alot of noise (low S/N). Only the peaks for Zn (0) species at binding energies around 1044.8 eV (Zn 2p_1/2_) and 1021.7 eV (Zn 2p_3/2_) are present after some light Ar^+^ sputtering. This shows that pyridine thiol forms a relatively thick surface layer. A summary of the XPS results and peak assignments can be found in the Supplementary Materials (Table S4).

### 3.8. UV-Vis Spectroscopy

**Figure 14:**
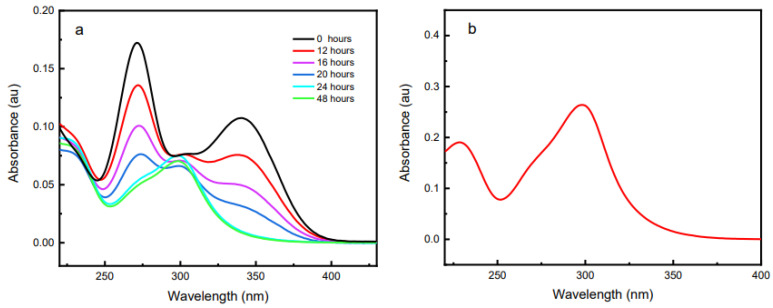
UV-vis spectroscopy of (**a**) P2T inhibited immersion solution over different time intervals and (**b**) UV-Vis spectrum of pure dipyridyl disulfide in 0.5 M H_2_SO_4_ solution.

**Figure 15:**
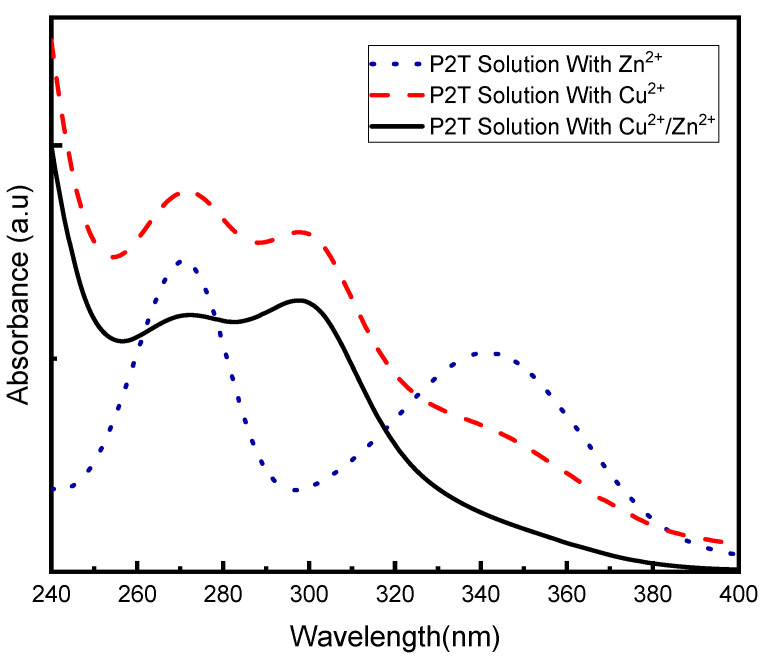
UV-vis spectroscopy of immersion solutions recorded after 24 h containing only Cu^2+^ ions (red dashed line); only Zn^2+^ ions (blue dotted line); or equal amounts of both Cu^2+^ and Zn^2+^ ion (black solid line).

UV-vis spectroscopy was used in order to research structural information of the inhibitor during the corrosion inhibition process. Figure 14a shows the UV visible spectrum of the corrosive medium containing P2T (with brass coupon in solution) over time. In the beginning pyridine thiol exhibits two peaks around 271 and 341 nm but after 12 h a new peak appears around 298 nm. Over time the height of the P2T peaks diminishes while the peak at 298 nm increases. After 24 h, peaks at 271 and 341 nm completely disappear and the peak at 298 nm remains the only visible peak. This suggests there is a structural change of the inhibitor taking place during the immersion period due to the oxidation of pyridine thiol to dipyridyl disulfide. Figure 14b shows the UV-vis spectrum of pure dipyridyl disulfide in 0.5 M H_2_SO_4_ and it is identical to the oxidation product of the P2T solution after 24 h. This has been observed in a few other studies as well [57,58]. The oxidation can be due to either aerobic oxidation by atmospheric oxygen or redox coupled reaction with the metal surface. The UV-vis spectra of P2T in 0.5 M H_2_SO_4_ without brass was also recorded (see Supplementary Materials Figure S2) and after 24 h no structural change was detected. This eliminates the possibility of aerobic oxidation and should be due to a redox reaction with the metal surface. To investigate the oxidation of pyridine-2-thiol, three separate immersion solutions of P2T dissolved in 0.5M H_2_SO_4_ were prepared and not exposed to the metal. To the first immersion solution only Cu^2+^ ions were added; to the second solution only Zn^2+^ ions were added; and to the third solution equal amounts of both Cu^2+^ and Zn^2+^ were added. UV-vis spectra of each solution were recorded after 24 h. Figure 15 displays the recorded UV-vis spectra and interestingly, the solution with only Zn^2+^ ions show UV peaks characteristic of P2T. However, in the case of the other two solutions, where Cu^2+^ ions are present, the structural change that was previously observed is seen with the disulfide species peaks appearing. The new peak around 300 nm appears after 24 h of immersion: meaning P2T becomes oxidized to the disulfide compound. This confirms the most likely mechanism of oxidation comes from the Cu^2+^ ions present during corrosion.

Previous studies have shown that Cu (II) species can react fast with thiols to form subsequent disulfides, but in this case, reaction is transpiring over 12–24 h [58,59]. Since pyridine thiol slows corrosion and stops the oxidation of copper, we believe initially there is not sufficient Cu (II) species to oxidize pyridine thiol. As time progress more Cu (II) species are added to solution and then oxidize pyridine thiol to dipyridyl disulfide. We propose the following mechanism as a possible reaction pathway.

Cu (II) + RSH → Cu (I) + RS·(12)

RS·+ RS·→ RSSR(13)

It is interesting to observe that after 12 h of immersion, both pyridine thiol and dipyridyl disulfide is present in the solution. Thus, corrosion inhibition may proceed through a mixed film formation on brass surface. Other researchers have found that dipyridyl disulfide can be used as an effective corrosion inhibitor agent against copper corrosion in acidic solutions. According to the AAS data, even after 48 h, the inhibitor provides some degree of corrosion protection and UV-Vis data confirms that after 24 h this protection is coming from the oxidation byproduct, dipyridyl disulfide.

A proposed mechanism for P2T adsorption on brass based on the adsorption isotherm studies, XPS data, and UV-vis spectra is mainly by the chemisorption process. The chemisorption process happens through direct covalent bond formation between copper and SH end of the thiol molecule as seen in XPS studies. XPS also shows that the nitrogen atom in the heterocyclic ring, at low pH is protonated and does not participate in the adsorption process. With time, inhibitor molecules can desorb back to the solution due to the corrosion attack and corrosion protection can become weakened significantly. However, in the case of P2T, even after 48 h of immersion there is significant protection from corrosion attack as seen in the AAS data. This can be due to the adsorption of the oxidation generated dipyridyl disulfide molecules onto the alloy surface as explained in the UV-vis analysis.

## 4. Conclusions

In this study, the corrosion protection nature of pyridine-2-thiol for brass in 0.5 M H_2_SO_4_ was studied. Potentiodynamic polarization data revealed that addition of corrosion inhibitor to the corrosive medium significantly reduced the i_corr_ values leading towards higher inhibition efficiencies. Adsorption behavior of P2T follows the Langmuir adsorption isotherm, and it is predominately chemisorption. Electrochemical impedance spectroscopy showed an increasing trend for the Rp and Rct values with increasing amount of corrosion inhibitors in the solution, where there is an optimum P2T concentration of 0.25 mM. XPS data confirmed that pyridine thiol actually adsorbs on to the surface forming a S-Cu bond. UV-vis spectroscopy reveals that over time the inhibitor becomes oxidized to dipyridyl disulfide, but the resulting compound is an effective corrosion inhibitor as well.

## Figures and Tables

**Table 1:** Calculated weight loss, corrosion rate, and inhibition efficiency without and with various concentrations of P2T.

[Inhibitor] (mM)	Weight Loss of Metal (mg)	Corrosion Rate (gm^−2^h^−1^)	Inhibitor Surface Coverage (Ɵ)	Inhibition Efficiency (µ %)
Blank	83.8 (±5)	0.73		-
0.05	80.1 (±4)	0.70	0.05	4.5
0.10	74.0 (±2)	0.64	0.11	11.7
0.25	60.7 (±4)	0.53	0.28	27.5
0.50	46.5 (±6)	0.40	0.45	44.5

**Table 2:** Electrochemical parameters (*E_corr_*, *i_corr_*, βa, βc) extrapolated from potentiodynamic polarization curves of brass in 0.5 M H_2_SO_4_ with and without corrosion inhibitor.

[Inhibitor] (mM)	*E_corr_* (mV)	*I_corr_* (µAcm^−2^)	β_a_ (mV/dec)	β_c_ (mV/dec)	Surface Coverage (Ɵ)	Inhibition Efficiency (µ%)
Blank	−80 (±5)	25.9	41.1	530.9	-	-
0.05	−85 (±4)	7.7	74.6	123.1	0.70	70
0.10	−98 (±9)	6.4	77.3	146.4	0.75	75
0.25	−212 (±16)	1.8	103.8	93.7	0.93	93
0.50	−167 (±11)	3.9	101.2	213.6	0.84	84

**Table 3:** Impedance parameters obtained by fitting EIS data using Zview software for the blank and in the presence of different concentrations of corrosion inhibitors.

Concentration (mM)	R_s_ (Ω cm^2^)	R_f_ (Ω cm^2^)	*CPE*_f_		R_ct_ (Ω cm^2^)	*CPE*_dl_		W (Ω cm^2^)
			T × 10^−6^ (Ω^−1^s^α^ cm^−2^)	n		T × 10^−6^ (Ω^−1^s^α^cm^−2^)	n	
Blank	5.76 (±0.37)	-	-	-	224 (±25)	186.2	0.85	214 (±115)
0.05	5.35 (±0.52)	135 (±23)	46.2	0.93	1477 (±113)	130.4	0.66	69 (±35)
0.10	4.9 (±0.33)	175 (±46)	37.9	0.96	1951 (±295)	39.2	0.69	110 (±47)
0.25	5.6 (±0.61)	1691 (±85)	29.2	0.97	4238 (±571)	18.4	0.80	-
0.50	4.7 (±0.45)	1184 (±117)	34.4	0.98	2800 (±467)	27.1	0.87	-

## Data Availability

The data presented in this study are available upon request from the corresponding author.

## Supplementary Materials

The following supporting information can be downloaded at: https://www.mdpi.com/article/10.3390/molecules27196550/s1, Table S1: Calculated inhibition efficiencies using zinc concentrations; Table S2: Calculated inhibition efficiencies using zinc concentrations; Table S3: Calculated capacitance values in the presence of different concentrations of corrosion inhibitors; Figure S1: Adsorption isotherm fitting for various models using the potentiodynamic polarization data at 25 °C Table S4: XPS Spectra comparison for inhibited treated brass; Figure S2: UV-vis spectroscopy of P2T inhibited immersion solution in 0.5 M H_2_SO_4_ solution without the presence of brass (citation: [44,60]).
