# Supplementary material for: Corrosion Inhibition Effect of Pyridine-2-Thiol for Brass in An Acidic Environment
Source: Molecules. 2022 Oct 3;27(19):6550. doi: 10.3390/molecules27196550 (PMC9573544; doi:10.3390/molecules27196550)
Supplement: Supplementary file 1 [file molecules-27-06550-s001.zip › molecules-1907238-supplementary.pdf]

Supplementary Data

Corrosion inhibition effect of pyridine-2-thiol as a corrosion inhibitor for brass in acidic environment

Darshan Jayasinghe Karunarathne, Alireza Aminifazl, Tori E. Abel, Karen L. Quepons, Teresa D. Golden*

Department of Chemistry, University of North Texas, 1155 Union Circle #305070, Denton, Texas 76203, USA

*Corresponding Author: Dr. Teresa D. Golden, tgolden@unt.edu, 940-565-2888

Table of Contents

Table S1: Calculated inhibition efficiencies using zinc concentrations……………………………………S2

Table S2: Calculated inhibition efficiencies using zinc concentrations………………………………..S2

Table S3: Calculated capacitance values in the presence of different concentrations of corrosion inhibitors…………………………………………………………………………………………………….S3

Figure S1: Adsorption isotherm fitting for various models using the potentiodynamic polarization data at 25°C…………………………………………………………………………………………S5

Table S4: XPS Spectra comparison for inhibited treated brass…..…………………………………………S6

Figure S2: UV-vis spectroscopy of P2T inhibited immersion solution in 0.5 M H_2SO_4 solution without the presence of brass…………………………………………………………………………………S7

Atomic Absorption Spectroscopy: Inhibition Efficiency Calculation

Inhibition efficiency using AAS can be calculated using both zinc concentration and the copper concentration. Following equation is used to calculate the inhibition efficiencies:

$$IE\% = \frac{C_{blank} - C_{inh}}{C_{blank}} \times 100\% \qquad (1)$$

C_{blank} is the concentration of the interested ion in the blank solution and C_{inh} is the concentration of interested ion in inhibited solution.

Table S1. Calculated inhibition efficiencies using zinc concentrations from AAS data.

**Concentration (mM)**	**IE%**
0.05	5.3
0.10	20.7
0.25	26.0
0.50	51.3

Table S2. Calculated inhibition efficiencies using copper concentrations from AAS data.

**Concentration (mM)**	**IE%**
0.05	3.6
0.10	26.1
0.25	28.2
0.50	42.5

Electrochemical Impedance Spectroscopy

The values for Cdl and Cf can also be calculated using a formula proposed by Brug et al. [1].

$$C = Rp^{(1-n)/n} \times Yo^{1/n} \quad (2)$$

Where Rp is the polarization resistance, Yo is the value of CPE and n is the deviation parameter.

Table S3. Calculated capacitance values in the presence of different concentrations of corrosion inhibitors.

Concentration (mM)	Cf (µF)	Cdl (µF)
Blank	-	106.3
0.05	31.5	55.8
0.10	30.7	12.4
0.25	26.6	9.7
0.50	32.2	20.5

A second theory suggested by Hsu and Mansfeld has the following equation and can be used to calculate the capacitance values using Wmax (maximum angular frequency) [2].

$$C = Yo\omega^{n-1} = Yo\left(2\pi f_{z_{im-Max}}\right)^{n-1} \quad (3)$$

Where $f_{z_{im-Max}}$ is the frequency at the maximum point of the imaginary part of the Nyquist plot. These formulas gave different values for capacitance values and Huang et al. proved that Brug formula yielded more accurate values than Hsu and Mansfled equation. Therefore we will use the values obtained from Brug equation for further analysis.

1. Brug, G. J.; Van Den Eeden, A L G; Sluyters-Rehbach, M.; Sluyters, J. H. The analysis of electrode impedances complicated by the presence of a constant phase element. Journal of Electroanalytical Chemistry and Interfacial Electrochemistry 1984, 176, 275.

2. Hsu, C. H.; Mansfeld, F. Technical Note: Concerning the Conversion of the Constant Phase Element Parameter Y0 into a Capacitance. Corrosion (Houston, Tex.) 2001, 57, 747-748

Adsorption isotherms

The adsorption process of an organic inhibitor to a metal surface from bulk solution can be simplified as the substitution of adsorbed water molecules on the metal surface by the organic corrosion inhibitor.

The following equation illustrates this mechanism.

$Org(sol) + xH2O(ads) \approx Org(ads) + H2O(sol)$ subscript?? (4)

In this equation Org(sol) and Org(ads) are the dissolved organic inhibitor in the bulk solution and adsorbed organic inhibitors. H_2O(ads) is the adsorbed water molecules on the metallic surface and x is the number of water molecules replaced by one molecule of organic corrosion inhibitor.

The following equations represent each isotherm that was used to fit the data and Figure XX represents the obtained fitting data.

Langmuir isotherm: $\frac{C}{\Theta} = \frac{1}{K} + c$ (5)

Temkin isotherm: $exp^{(-2\alpha\Theta)} = KC$ (6)

Frumkin isotherm: $ln\left[\frac{\Theta}{(1-\Theta)C}\right] = lnK + 2\alpha\Theta$ (7)

Flory-Huggins isotherm: $ln\frac{\Theta}{C} = xln(1-\Theta) + \ln(xKads)$ (8)

El-Awady isotherm: $ln\frac{\Theta}{1-\Theta} = ylnC + lnK'$ (9)

Freundlich isotherm: $log\Theta = nlogC + logKads$ (10)

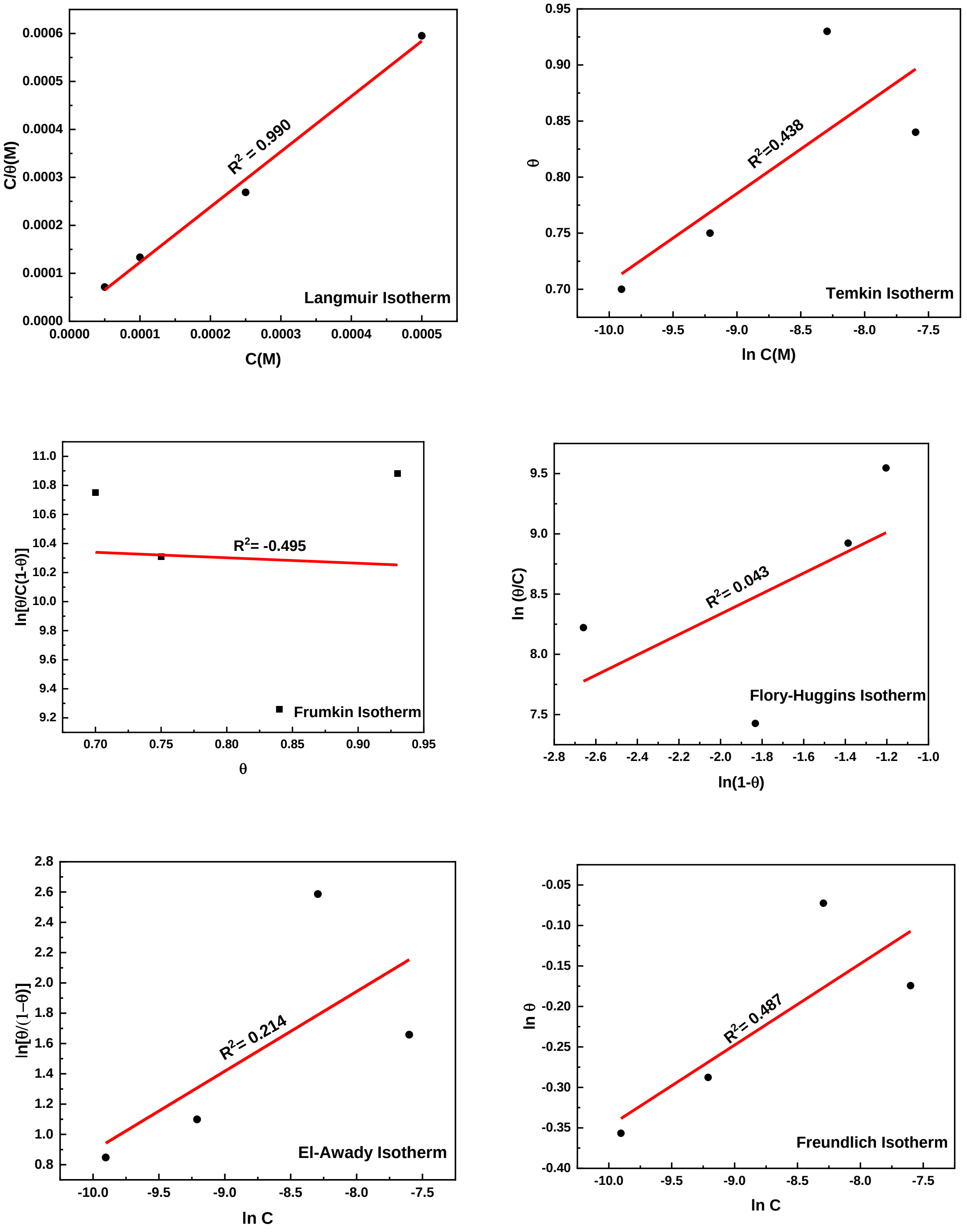

Figure S1 Adsorption isotherm fitting for various models using the potentiodynamic polarization data at 25^oC.

X-ray photoelectron Spectroscopy

Table S4: XPS Spectra Comparison for Inhibited treated brass.

Element of interest	XPS Peak	Chemical Bonding	Peak values from this Study (eV)
Carbon	C 1S	C-C / C-H	284.8<br><br>286.5
Sulfur	S 2p	S-Cu	162.2<br><br>168.3
Nitrogen	N 1S	N-H+	400.3
Oxygen	O 1S	Cu_2O	530.8
Copper	Cu 2p3/2<br><br>Cu 2p1/2	Cu(0)/Cu(+)<br><br>Cu(0)/Cu(+)	932.2<br><br>952.1
Zinc	Zn 2p3/2<br><br>Zn 2p1/2	Zn(0)<br><br>Zn(0)	1021.7<br><br>1044.8

UV-vis Spectroscopy

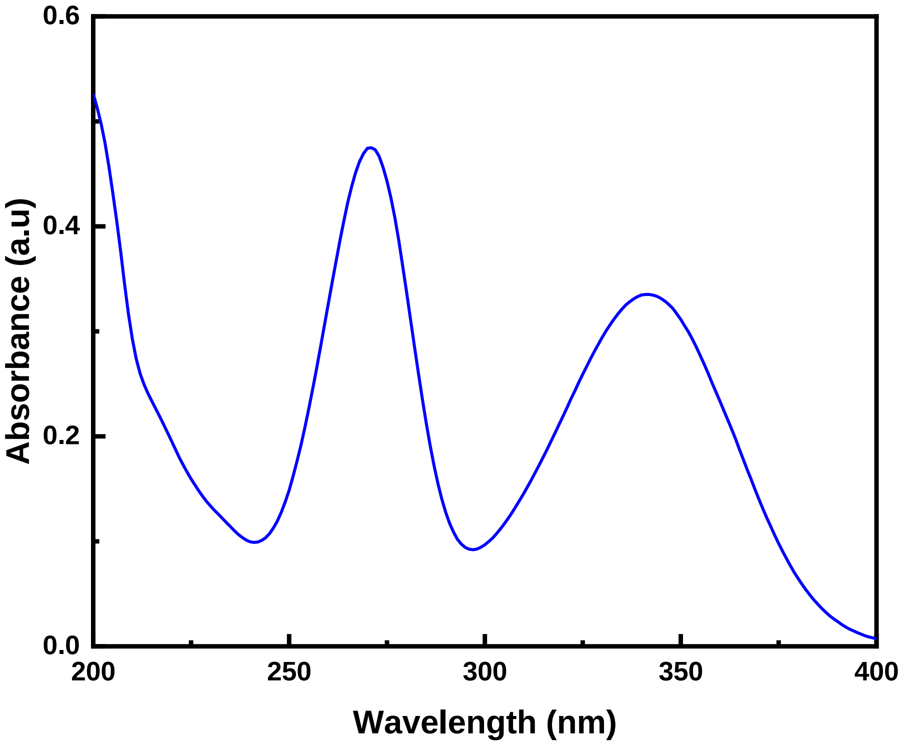

Figure S2. UV-vis spectroscopy of P2T inhibited immersion solution in 0.5 M H_2SO_4 solution without the presence of brass after 24 hours.
